# Integrating hypertension detection and management in HIV care in South Africa: protocol for a stepped-wedged cluster randomized effectiveness-implementation hybrid trial

**DOI:** 10.1186/s43058-024-00640-6

**Published:** 2024-10-14

**Authors:** Karla I. Galaviz, Shivani A. Patel, Mark J. Siedner, Charles W. Goss, Siphamandla B. Gumede, Leslie C. Johnson, Claudia E. Ordóñez, Michael Laxy, Kerstin Klipstein-Grobusch, Martin Heine, Mary Masterson, Aaloke Mody, W. D. Francois Venter, Vincent C. Marconi, Mohammed K. Ali, Samanta T. Lalla-Edward

**Affiliations:** 1 Indiana University School of Public Health Bloomington , #1025 E 7th St, Bloomington, IN 47405 USA; 2 Hubert Department of Global Health, Emory University, Atlanta, GA USA; 3 Emory Global Diabetes Research Center of the Woodruff Health Sciences Center, Emory University, Atlanta, GA USA; 4 Africa Health Research Institute, KwaZulu-Natal, South Africa; 5 Harvard Medical School and Massachusetts General Hospital, Boston, MA USA; 6 Washington University School of Medicine in St. Louis, St. Louis, MO USA; 7 Ezintsha, Faculty of Health Sciences, University of the Witwatersrand, Johannesburg, South Africa; 8 Department of Family and Preventive Medicine, School of Medicine, Emory University, Atlanta, GA USA; 9 School of Medicine and Health, Technical University of Munich, Munich, Germany; 10 Department of Global Public Health and Bioethics, Julius Center for Health Sciences and Primary Care, University Medical Center Utrecht, Utrecht University, Utrecht, The Netherlands; 11 Division of Epidemiology and Biostatistics, School of Public Health, Faculty of Health Sciences, University of the Witwatersrand, Johannesburg, South Africa; 12 Institute of Sport and Exercise Medicine, Department of Exercise, Sport and Lifestyle Medicine, Faculty of Medicine and Health Sciences, Stellenbosch University, Cape Town, South Africa; 13 National Heart, Lung and Blood Institute, National Institutes of Health, Bethesda, MD USA; 14 Division of Infectious Diseases, Emory University School of Medicine, Atlanta, GA USA

**Keywords:** Public primary care, Cardiovascular disease, Comorbidities, Hypertension, HIV

## Abstract

**Background:**

HIV clinical guidelines recommend hypertension detection and management to lower cardiovascular disease risk, but these have not been effectively implemented for people living with HIV (PWH). Addressing this implementation gap requires community-engaged implementation studies focused on addressing implementation barriers specific to the HIV care context.

**Methods:**

This protocol describes a type 2 effectiveness-implementation hybrid study conducted in nine primary care clinics in Johannesburg. The study will evaluate the effect of implementation strategies on guideline-recommended blood pressure assessment and management in HIV clinics and the effects of assessment/management on patient blood pressure. A stepped-wedge, cluster randomized study design was used to randomize clinics to the time at which they receive the implementation strategies and patient intervention. The implementation strategies tested include identifying and preparing care champions, changing record systems, conducting ongoing training, providing audit and feedback, and changing the physical structure/equipment. The patient intervention tested includes detection of elevated blood pressure, educational materials, lifestyle modification advice, and medication where needed. Implementation outcomes include adoption, fidelity (co-primary outcome), cost, and maintenance of the blood pressure assessment protocol in participating clinics, while patient outcomes include reach, effectiveness (co-primary outcome), and long-term effects of the intervention on patient blood pressure. These will be assessed via direct observation, study records, staff logs, medical chart reviews, and patient and healthcare worker surveys. To examine effects on the implementation (intervention fidelity) and effectiveness (patient blood pressure changes) co-primary outcomes, we will use the standard Hussey and Hughes model for analysis of stepped-wedge designs which includes fixed effects for both interventions and time periods, and a random effect for sites. Finally, we will examine the costs for the implementation strategies, healthcare worker time, and patient-facing intervention materials, as well as the cost-effectiveness and cost-utility of the intervention using study records, patient surveys, and a time and motion assessment.

**Discussion:**

This study will address knowledge gaps around implementation of cardiovascular disease preventive practices in HIV care in South Africa. In doing so, it will provide a dual opportunity to promote evidence-based care in the South African HIV care context and help refine implementation research methods to better serve HIV populations globally.

**Trial registration:**

ClinicalTrials.gov: NCT05846503. Registered on May 6, 2023. https://classic.clinicaltrials.gov/ct2/show/NCT05846503.

**Supplementary Information:**

The online version contains supplementary material available at 10.1186/s43058-024-00640-6.

Contributions to the literature

- This protocol describes the evaluation of context-specific implementation strategies to improve the detection and management of hypertension in HIV care settings in Johannesburg, South Africa.
- Implementation strategies identified through community-engaged formative work are used to promote guideline-recommended hypertension detection and management in routine HIV care.
- The evaluation employs robust implementation research methods that can produce internally and externally valid findings to inform integration of guideline-recommended hypertension care practices into routine HIV care.

## Background

Widespread availability of antiretroviral therapy has extended survival among people with HIV (PWH) such that age-related chronic conditions are growing in prevalence for this population [1–5]. Notably, the cardiovascular disease (CVD) burden for PWH has tripled over the past two decades, accounting for 2.6 million disability-adjusted life-years lost each year globally [6]. South Africa has become an epicenter of the dual burdens of infectious and non-communicable diseases, with the highest absolute number of PWH worldwide [7] and growing burdens of CVD [8–10]. Addressing the dual burden of HIV infection and CVD risk factors faced by PWH in South Africa requires integrated care models that account for financial and human resources limitations present in the healthcare system [11].

Evidence-based recommendations endorse hypertension detection and management to lower CVD risk in PWH [12], but these have not been effectively implemented for this population [13–15]. Studies have shown hypertension management is suboptimal in HIV care [16, 17], and that PWH are less likely to receive guideline-recommended CVD preventive care than those without HIV [18]. Identified challenges to implementing evidence-based hypertension management include lack of investments for integrating HIV and CVD care, lack of standardized screening practices, and lack of validated care algorithms [19, 20]. In addition, the PEPFAR and Global Fund strategies have often led to vertically siloed HIV care models wherein other health conditions are addressed in separate settings. Overcoming these challenges requires community-engaged implementation studies focused on addressing barriers to guideline implementation specific to HIV care settings.

Based on our community-engaged formative work [21], we designed context-specific implementation strategies to improve hypertension detection, treatment, and control among PWH in South Africa. Here, we describe the protocol of a type 2 effectiveness-implementation hybrid study aimed at evaluating the effects of our implementation strategies and clinical intervention in nine primary health care clinics in the city of Johannesburg, South Africa. To inform decision-makers of the potential costs associated with integrating hypertension detection and control in HIV care, we also describe the protocol for evaluating budget impact and cost-effectiveness of this multi-strategy approach.

## Methods

### Setting

The study is being conducted in public sector primary care clinics that provide HIV care in Region F of Johannesburg, South Africa. The region has 14 primary care clinics that serve about 700,000 residents by offering reproductive and sexual health care, mobile and community outreach services, HIV screening and treatment, and tuberculosis screening and treatment. Prior to this study, two clinics also had hypertension management programs in place. A mix of patients with and without HIV are seen in these clinics, and a high proportion of patients are non-native migrants (> 75%). Primary care services are led by nurses, while HIV diagnosis and treatment services are led by nurses trained in the Nurse Initiation and Management of Antiretroviral Therapy protocol. All 14 primary care clinics were invited to participate in the study: four declined (due to renovations, limited staff, or unsuitable patient care flow), and one clinic was selected for a pilot study. The remaining nine clinics agreed to participate and were enrolled in the study.

### Study design

 We employ a type 2 effectiveness-implementation hybrid study type to simultaneously test implementation strategies and a clinical intervention [22]. Specifically, we will evaluate the effect of the implementation strategies on guideline-concordant hypertension care practices in participating clinics, as well as the effects of the guideline-concordant care on patient blood pressure. For the evaluation, we employ a stepped-wedge, cluster randomized trial design. This protocol aligns with the Standards for Reporting Implementation Studies Statement [23] and with the CONSORT statement for reporting stepped-wedge, cluster randomized trials [24]. The study protocol is registered in ClinicalTrials.gov (NCT05846503).

We selected a stepped-wedge, cluster randomized trial design for three reasons. First, since the study has the potential to improve healthcare delivery, all participating clinics wanted to receive the intervention and this design ensures all clinics do. Second, this is an implementation study testing interventions under routine care conditions; as such, a sequential roll out of the proposed intervention allowed us to alter care for a proportion of clinics at a time. Finally, given the research and clinical resources available, rolling out the intervention in all clinics at the same time (i.e., cluster RCT) was not feasible; thus, a sequenced rolled out was preferred. These reasons outweighed the main limitations of the clustered randomized stepped wedge study design – selection bias and confounding due to temporal changes in clinical care concurrent with the intervention rollout [25].

Table 1 depicts the trial design, periods, and sequence allocation order. In our study, clinic was the unit of randomization. Clinics were stratified into three groups based on clinic population size, categorized by monthly patient head count as small (1000 to 3499), medium (3500 to 6999), and large (7000 to 10000). Clinics were then randomized via a computer-generated sequence to cross-over from the control to the intervention condition starting in Period 2 until all clinics crossed over to intervention. One clinic from each size stratum was randomly selected for each sequence. The study biostatistician (CWG) generated the randomization schedule and assigned clusters to sequences using SAS software, version 9.4 (SAS Institute Inc, NC USA). SLE enrolled the clusters.

The allocation sequence began with an observation period where none of the clinics had neither received the implementation strategies nor rolled out the patient intervention (Period 1). In this period, we collected implementation and clinical data under the control condition. After the control period, clinics crossed-over to the intervention condition, three at a time based on their randomization sequence. To date, all nine clinics have-crossed over to the active intervention period which will run from study months 4–15 (Periods 2–5). Since this intervention was randomized at the clinic level and involves providing clinical care, blinding was not possible. For each step, clinics were notified one month in advance prior to initiation of the intervention.

At month 14, all clinics will initiate a two-month offboarding transition period (Period 5). Here, research staff will support clinic staff to ensure that implementation strategies continue to be executed, while also helping them to further integrate the blood pressure assessment protocol within routine care. From months 16–27, research staff will be withdrawn from clinics and a maintenance observation period will begin (see Table 1).

**Table 1:**
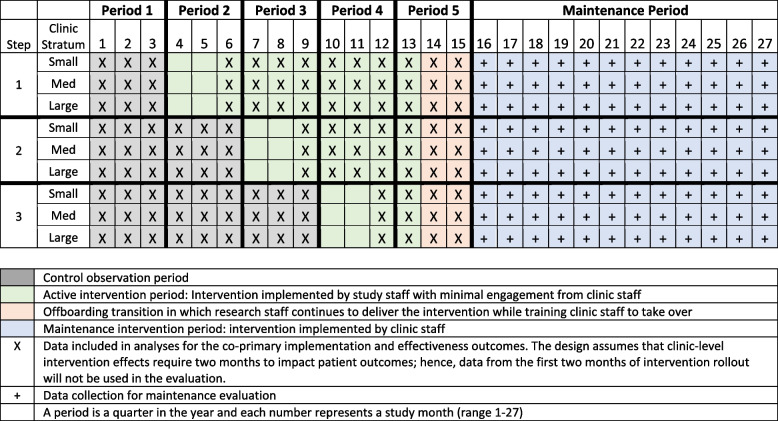
Trial design and random sequence allocation order

### Study populations

**Fig. 1:**
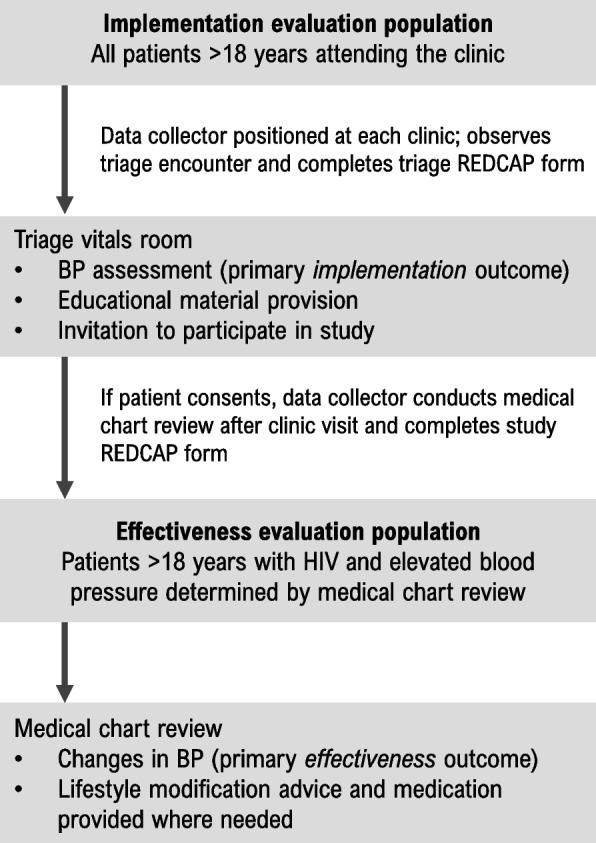
Implementation and effectiveness evaluation populations selection flow. BP = blood pressure

 The study includes two analytic study populations: the implementation evaluation and the effectiveness evaluation population. Figure 1 shows the implementation and effectiveness population selection flow. The implementation evaluation population will include all patients ≥ 18 years old who present to clinic for care and are seen in the study triage room where vital signs are taken. Data from this population will be collected by a research staff member situated in the triage room who will record whether blood pressure is measured, the blood pressure values obtained, and whether educational materials are delivered at each visit. These data will be recorded using a REDCAP form developed for the study. These patients will contribute data to the primary implementation outcome for all study steps, including control and intervention periods.

The effectiveness evaluation population will include patients aged ≥18 years old with elevated blood pressure (defined as systolic blood pressure ≥140 mmHg or diastolic blood pressure ≥90 mmHg), at any point during the active intervention period, and with a known diagnosis of HIV (determined by self-report or medical chart review). Patients are eligible regardless of their prior history of hypertension or use of blood pressure lowering medications. Research staff situated in the triage vitals room will recruit these patients and obtain consent to prospectively abstract demographic and clinical data from their medical charts. Patients will be included in the study through continuous recruitment. Patients with HIV and elevated blood pressure then become part of the effectiveness outcome population from the date of their first elevated blood pressure value is recorded and will remain under observation for all additional study visits.

### Implementation strategies

The implementation strategies used in this study aim to promote fidelity to the hypertension detection and treatment protocol recommended by the South African Hypertension Society Guidelines [26]. The implementation strategies were designed through community-engaged formative work that was guided by the Behavior Change Wheel [27]. Details and results from our formative work are reported elsewhere [21]. Briefly, we conducted interviews with relevant actors (patients, clinicians, and administrators) to identify barriers to detecting and treating elevated blood pressure (for healthcare workers), or to managing it (for patients). The barriers identified were reviewed and ranked by the study’s Community Advisory Board and a final set of barriers to be addressed in the study was selected. These barriers were then mapped onto relevant intervention functions and behavior change techniques specified in the Behavior Change Wheel [27].

Four implementation strategies resulted from our formative work: identify and prepare care champions, change record systems, conduct ongoing training, and audit and provide feedback. Because blood pressure assessment equipment was lacking in participating clinics, a fifth implementation strategy was added –changing the physical structure and equipment. The five implementation strategies tested in this study are described in Table 2. These were defined using Expert Recommendations for Implementing Change [28] and specified according to established recommendations [29]. The implementation strategies will be carried out by the study care coordinator during the active intervention period. During the maintenance period, the care coordinator will be withdrawn, and the implementation strategies will be carried out by clinic staff.

**Table 2:** Implementation strategy specification

Name	Specification^a^ (actor, action, target)	Temporality and dose
Identify and prepare care champions	Care coordinator recruited and trained to assist healthcare workers with implementation of the elevated blood pressure detection and treatment protocol.	Throughout active intervention period
Change record systems	Information management system that includes a dashboard summarizing blood pressure data each month (e.g., case detection and treatment rates), and patient flow charts to record data on blood pressure assessment results and treatment. The dashboard is updated by research staff and presented by the care coordinator at a monthly clinic meetings.	Throughout active intervention period
Conduct ongoing training	Ongoing training sessions for healthcare workers delivered by the care coordinator on elevated blood pressure detection and treatment, data interpretation, and patient-provider communication skills.	Monthly: during active intervention period
Change physical structure and equipment	Dinamap™ and portable blood pressure assessment machines placed at participating clinics used and maintained by the care coordinator.	At intervention rollout
Audit and provide feedback	Sessions delivered by the care coordinator where the dashboard data for a given month is presented to healthcare workers, clinic managers, and staff. Programmatic feedback is provided to discuss how to improve case detection and treatment.	Monthly: during active intervention period

^a^During the maintenance period, the care coordinator (actor) will be withdrawn, and all the activities specified in each implementation strategy will be carried out by clinic staff

### Clinical intervention

**Fig. 2:**
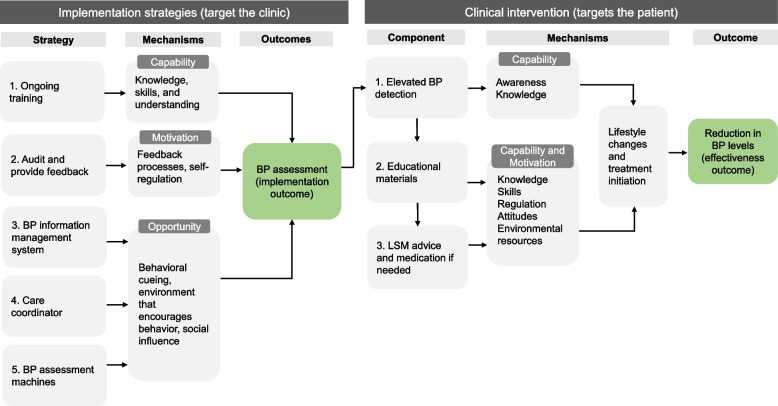
Intervention theory depicting the hypothesized action mechanisms and outcomes for the implementation strategies and the clinical intervention. BP = blood pressure; LSM = lifestyle modification

The clinical intervention follows the recommendations from the South African Hypertension Society Guidelines [26] and includes the following components: (1) assessing blood pressure, (2) providing patient education, (3) providing brief lifestyle modification advice and, where needed, (4) providing blood pressure lowering medication (Fig. 2). Patient educational materials include color-coded posters placed in clinics and individual pocket-sized booklets explaining blood pressure levels (green = normal, yellow = high/elevated, red = very high), and what to do to lower or maintain blood pressure levels. Booklets also provide a longitudinal record for patients to self-monitor their blood pressure levels over time. All materials are available in English, isiZulu, isiXhosa, and Shona to cater for most of the populations clinics serve. All patients in these clinics receive this intervention regardless of the purpose of the visit or medical history unless they attend during the control period and do not return to the clinic.

### Outcome measures and definitions

Our study outcomes are based on the RE-AIM framework [30]. The two primary outcomes are intervention fidelity to the blood pressure assessment protocol in clinics (*implementation*) and changes in blood pressure in patients with elevated values (*effectiveness).* Secondary outcomes include *adoption*,* cost* and *maintenance* of the blood pressure assessment protocol in clinics, as well as the *reach* and effect *maintenance* of the intervention among patients. Data for these outcomes will be obtained from patient medical charts, direct observation, REDCAP [31] data collection forms designed for this study, patient surveys, study records, and healthcare worker surveys. The RE-AIM outcomes, their assessment time points, and their data sources are reported in Table 3.

**Table 3:** RE-AIM outcome definitions, data sources and data collection time points

**IMPLEMENTATION EVALUATION**		
**Outcome definitions **	**Time points**	**Data source**
***Adoption*** Participation rate and representativeness of clinics that initiate the blood pressure assessment intervention	At every initial study step (i.e., just the initial rollout step)	• Study REDCAP form
***Implementation Fidelity*** Primary implementation outcome: extent to which the BP measurement protocol is implemented	All clinic visits in months 1–15, excluding the two months following intervention initiation (during research staff implementation phase)	• Medical charts • Study REDCAP form • Adaptations recorded by care coordinator / research staff
***Cost*** Initial costs of implementation strategies and cost of implementing the BP measurement protocol	Control, intervention (months 1–15) and maintenance (months 16–27) periods	• Study records • Time and motion assessment
***Clinic-level Maintenance*** Extent to which BP continues to be measured and is normalized after active intervention period	Months 16–27 (during clinic staff implementation)	• Medical charts • Healthcare worker normalization questionnaire
**EFFECTIVENESS EVALUATION**		
**Outcome definitions**	**Time points**	**Data source**
***Reach*** Percentage and representativeness of patients with elevated blood pressure and HIV that receive the intervention	At the initial / enrollment study visit (months 1–15)	• Study REDCAP form • Medical charts
***Effectiveness*** Primary clinical outcome: difference in mean systolic BP between the intervention and control periods	All visits in months 1–15, excluding the two months following intervention initiation	• Medical charts
***Cost-effectiveness*** Health utility of the intervention and direct medical costs of additional patient visits and/or hospitalizations	Control, active intervention (months 1–15), and maintenance periods (months 16–27)	• Quality of life (EQ-5D) patient questionnaire • Patient-reported cost survey • Study records
***Patient-level Maintenance*** Extent to which blood pressure improvements observed during the intervention phase are maintained	Active intervention (months 4–15) and maintenance (months 16–27) periods	• Medical charts • Study REDCAP form

*BP* blood pressure

*Adoption* will be determined as the percentage and representativeness of clinics that complete the blood pressure assessment protocol at the initial study step (i.e., did they roll out intervention? Yes/No). Intervention fidelity (*primary implementation outcome*) will be defined as the difference in percentage of patient visits with recorded blood pressure measurement between intervention and control periods. We will also keep track of adaptations made to the blood pressure assessment protocol via care coordinator notes and reports. Clinic-level *maintenance* of the intervention will be defined as the extent to which blood pressure continues to be assessed after the active intervention period ends based on medical chart reviews. We will also assess the normalization of the blood pressure assessment protocol using a 23-item questionnaire [32] healthcare workers will complete at the beginning and at the end of the maintenance period.

*Reach* will be defined as the percentage and representativeness of patients with elevated blood pressure that receive educational materials and lifestyle modification advice / medication, among all patients with elevated blood pressure. Changes in patient blood pressure (*primary effectiveness outcome*) will be defined as the difference in mean systolic blood pressure between the intervention and control periods. Patient-level m*aintenance* of intervention effects will be defined as the extent to which blood pressure improvements observed during the active intervention period are sustained during the maintenance period.

The *cost* evaluation will examine direct medical costs for intervention delivery, including implementation strategies, patient-facing intervention materials, additional staff time required to deliver the intervention, and additional blood pressure medication. One-time costs for implementation strategies will be estimated from the study records. The additional time burden associated with integrating the blood pressure assessment protocol in participating clinics will be assessed using a time and motion assessment in a random sub-sample of 100 patients at each clinic during control, active intervention, and maintenance periods (900 per period; total *n* = 2,700). This assessment will collect data on the start and end time of different procedures during a patient’s journey through a routine clinic visit. Finally, this patient subsample will also complete a survey regarding medical expenditures for outpatient and inpatient care utilization during the last 12 months, and the EQ-5D quality of life questionnaire [33] to determine health utility.

### Sample size calculation

As a type 2 hybrid effectiveness-implementation study, this study has two primary outcomes – fidelity to the blood pressure assessment protocol and effectiveness of the protocol. Type 2 hybrid studies can be powered on either primary outcome [34] given that the primary outcomes belong to separate samples and different interventions. Thus, this study is powered to detect effectiveness of the clinical intervention (changes in mean systolic blood pressure comparing control with intervention periods). To detect a conservative difference in mean systolic blood pressure of 5 mmHg between patients under the intervention versus control periods, with 85% power and alpha = 0.05, assuming a standard deviation (SD) in systolic blood pressure of 20 mmHg (SD computed from population-based data of adults in India and USA [35, 36]) we would require a sample of 506 patients across all clinics, not accounting for the design effect [37]. Assuming an intra-class correlation coefficient of 0.01, three steps, and nine study clinics over 15 months, we estimated a design effect of 3.2 [37]. After applying the design effect, 1640 patients are required, amounting to 46 patients with HIV and elevated blood pressure during the study period who are seen at each of three steps per clinic, which corresponds to an average of 184 patients seen annually per clinic. Because the smallest clinic in our sample serves > 300 patients with HIV and hypertension monthly (1725 patients seen monthly; ~20% with hypertension), we anticipate sufficient power to test our primary effectiveness hypothesis.

### Statistical methods

#### Overview

The statistical analyses are described according to implementation outcomes derived from clinics (i.e., *adoption*,* implementation*, and *maintenance* of the blood pressure assessment protocol) and outcomes related to intervention impact (i.e., *reach*,* effectiveness*, and *effect maintenance* of the intervention). We will use the standard Hussey and Hughes model for analysis of stepped-wedge designs [38] which includes fixed effects for both interventions and time periods, and a random effect for sites. We will extend this model to account for non-normal outcomes, repeated measurements on participants, and to include key covariates for each RE-AIM outcome as described below. All outcomes, patient characteristics, and clinic characteristics will be summarized (both overall and by intervention status) using means and standard deviations for continuous variables (or median [IQR] if data are skewed) and frequency counts with percentages for categorical variables. Statistical differences between pre- and post-intervention and initiated versus delayed (control) clinics during each observation period will be examined. Contingent on the observed distributions, differences in categorical outcomes will be assessed using chi-square or Fisher’s exact tests, and differences in continuous variables will be tested using independent group t-tests or Wilcoxon rank-sum tests. *P*-values < 0.05 will be considered statistically significant. Following these analyses, the costing, cost-effectiveness, and budget impact analysis will be conducted and are described at the end of this section.

#### Implementation outcomes analyses

To assess fidelity of the blood pressure assessment protocol (*primary implementation outcome*) we will examine the difference in percent of patient-visits with recorded blood pressure (1 = blood pressure measured; 0 = blood pressure not measured) between intervention and control clinics using mixed effects log-binomial regression. If the mixed effects log-binomial model fails to converge, we will use a generalized estimating equation (GEE) log-binomial model with exchangeable correlation structure and robust standard errors; if the GEE log-binomial fails to converge, we will use a GEE log-Poisson model [39, 40]. We will examine changes in blood pressure assessment between clusters (“horizontal”) and across periods (“vertical”) to evaluate heterogeneity in treatment effects by cluster and period, respectively. Between-cluster and between-period heterogeneity will be evaluated through an intervention by clinic interaction term.

Adaptation data recorded during the active intervention period will be examined using means and standard deviations (or median [IQR] if data are skewed) and frequency counts with percentages. Maintenance of blood pressure assessment by clinic staff will be examined by adding an interaction term between intervention exposure status (control vs. intervention) and implementation phase (i.e., research staff vs. clinic staff) to the model described for the primary implementation outcome. Means and standard deviations (or median IQR) will be used to summarize normalization process questionnaire [32] data at the beginning and the end of the maintenance period. Paired samples t-tests (or Wilcoxon rank-sum tests) will be used to compare normalization questionnaire scores at the beginning and the end of the maintenance period.

Exploratory analyses will examine exposure to the implementation strategies (e.g., > 80% of audit and feedback sessions received) and compare blood pressure assessment rates against that of clinics with lower exposure via the modeling approach for dichotomous outcomes previously described. Causal diagrams and latent variable modeling will also be considered as exploratory tools to examine mechanisms through which implementation strategies affect blood pressure assessment implementation.

#### Effectiveness outcomes analyses

To assess *reach*, we will summarize participant-level indicators, including age, sex, blood pressure at enrolment, treatment for blood pressure at enrolment, and HIV history by clinic at the initial intervention visit. We will then estimate the percentage of patients with elevated blood pressure that receive the initial educational materials and lifestyle advice plus medication (numerator), from all patients identified with elevated blood pressure (denominator). Demographic and clinical characteristics between reached/not reached patients will be compared using chi-square / Fisher’s exact tests for categorical variables, and independent samples t-tests / Wilcoxon rank-sum tests for continuous variables.

To assess clinical impact of the intervention (*primary effectiveness outcome)*, we will examine differences in mean systolic blood pressure between intervention versus control clinics. We will estimate the effect of clinic-level intervention exposure (no intervention versus intervention by research staff) using mixed effects linear regression models. In addition to the standard stepped-wedge model outlined previously, we will include a random effect to account for correlation due to repeated measurements on participants. We will also include randomization strata, sociodemographic characteristics (age, sex, and immigration status), and clinical characteristics (prior treatment for hypertension, HIV regimen, CD4 count, and viral load) in our final multivariable model to obtain an adjusted estimate of the intervention effect.

The effectiveness analysis will use data from months 1–15 while censoring data from the first two months of intervention initiation (i.e., the transition period from control to intervention; see Table 1). This is to provide time for the intervention to impact patient behavior and medication initiation, thus giving time for blood pressure to be affected. We will follow an intention-to-treat approach in which data from individual patients will be analyzed according to the clinic intervention status at the time of measurement. Standard model diagnostic procedures will be used to evaluate model fit, and transformations will be applied (e.g., log transformation) as needed.

Similar to the analyses described for the implementation outcome, we will evaluate treatment heterogeneity between clusters (“horizontal”) and across periods (“vertical”). Additional subgroup analyses will evaluate treatment-effect heterogeneity by prior treatment for hypertension, HIV regimen, CD4 count, viral load, age, sex, and immigration status. We will present subgroup analyses with *P*-values from interaction tests as well as estimates of mean differences and 95% confidence intervals. In exploratory analyses, we will use a longitudinal cohort approach restricted to patients with two or more visits and with moderate-to-severe hypertension (i.e., systolic blood pressure > 160 mmHg or diastolic blood pressure > 100 mmHg) during the active intervention period. We will apply linear or logistic models, contingent on the distribution of the outcome, following the approach described for the primary implementation outcome analyses.

To assess *maintenance* of clinical effects, we will determine if the blood pressure improvements observed during the active intervention phase (research staff implementation, months 4–15) differ from those observed in the maintenance phase (clinic staff implementation, months 16–27). For this, we will use the same linear mixed model described for the primary effectiveness outcome and estimate the mean difference in systolic blood pressure between active intervention and maintenance periods. Results will be presented as a mean difference and 95% confidence intervals. If the mean is higher in the maintenance period and the 95% CI does not overlap zero, we will conclude that the improvements on systolic blood pressure were not maintained.

#### Costing, cost-effectiveness and budget impact analyses

These analyses will concentrate on direct medical costs including costs for intervention delivery and costs for outpatient and inpatient health care utilization, taking roughly a public healthcare system perspective. Time of healthcare staff for implementing the intervention will be valued according to average gross salaries including fringe benefits. The change in hypertension medication cost will be monetarized by multiplying the volume of hypertension medication with average market prices for hypertension medication. We will describe raw absolute accrued costs for each cost category in the intervention and control periods and stratified for the control, active intervention, and maintenance period. Since there is no value set for South Africa, we will use an established scoring algorithm developed for a neighboring country [41] to translate EQ-5D health states into health utilities. We will assess cost-effectiveness as the incremental cost per mmHg reduction in systolic blood pressure and the cost-utility as the incremental cost per quality adjusted life year gained [42]. Uncertainty in incremental costs, utility, and cost-effectiveness/cost-utility will be assessed through non-parametric bootstrapping methods [43]. We will report the cost-effectiveness plane and the cost-effectiveness acceptability curve for different willingness to pay thresholds for a gain in one quality adjusted life year. We will also conduct rough extrapolations to assess the expected intermediate- and long-term budget impact on population level [44, 45]. For these analyses, we will follow the Consolidated Health Economic Evaluation Reporting Standards 2022 [46].

## Discussion

This study aims to examine whether context-specific implementation strategies improve the detection and management of elevated blood pressure among PWH in South Africa. We will test five implementation strategies designed through community-engaged formative work [21] using a type 2 effectiveness-implementation hybrid study [34] and a stepped-wedge cluster randomized design. These methods assure both internal validity for testing the effects of the implementation strategies and external validity by approximating the conditions of real-world HIV care. Finally, this study incorporates budget impact and cost-effectiveness analyses which will inform decision-makers of the potential costs associated with integrating guideline-recommended CVD preventive practices in HIV care.

Several policies and programs aimed at integrating non-communicable disease and HIV care have been rolled out in several Sub-Saharan African countries, but several challenges have hampered large-scale implementation [19, 20]. This study is among the first to systematically address implementation challenges in the South African HIV care system and will contribute evidence to inform large-scale integration of HIV-CVD care in this setting. Our study is part of a consortium of studies funded by the US National Heart Lung Blood and Sleep Institute focused on testing diverse implementation models to address CVD co-morbidities in PWH [47]. Another study that is part of this alliance is focused on testing practice facilitation to improve hypertension treatment in HIV care in Nigeria [48]. Similarly, a study in Mozambique is using a systems analysis and improvement approach to optimize the hypertension diagnosis and care cascade for PWH [49]. The studies in this alliance will contribute evidence on effective implementation models to promote the integration of HIV-CVD care in Sub-Saharan Africa.

This study employs diverse implementation research methods that ensure rigorous testing of our implementation strategies and advance implementation research in global settings. For instance, our study employs implementation theories, frameworks and methods to guide our formative work, intervention and strategy design, data collection, analyses, and interpretation. Hence, this study provides a dual opportunity to test these methods in the South African clinical context and learn about refinements needed for global settings and populations [50]. This study will also contribute to addressing implementation research gaps previously identified in HIV-CVD care integration in Sub-Saharan Africa [51] by employing a rigorous study design and reliable measures to answer research questions in low-resource, global contexts.

## Supplementary Information

Supplementary Material 1.

Supplementary Material 2.

## Data Availability

Data sharing is not applicable in this manuscript since no datasets were generated or analyzed.
